# Perpendicular alignment of lymphatic endothelial cells in response to spatial gradients in wall shear stress

**DOI:** 10.1038/s42003-019-0732-8

**Published:** 2020-02-06

**Authors:** Eleftheria Michalaki, Vinay N. Surya, Gerald G. Fuller, Alexander R. Dunn

**Affiliations:** 1 Dept. of Chemical Engineering, Stanford University, Stanford, CA 94305 USA; 2 Stanford Cardiovascular Institute, Stanford University School of Medicine, Stanford, CA 94305 USA

**Keywords:** Biophysics, Cell adhesion, Cell migration

## Abstract

One-way valves in the lymphatic system form from lymphatic endothelial cells (LECs) during embryonic development and are required for efficient tissue drainage. Although fluid flow is thought to guide both valve formation and maintenance, how this occurs at a mechanistic level remains incompletely understood. We built microfluidic devices that reproduce critical aspects of the fluid flow patterns found at sites of valvulogenesis. Using these devices, we observed that LECs replicated aspects of the early steps in valvulogenesis: cells oriented perpendicular to flow in the region of maximum wall shear stress (WSS) and exhibited enhanced nuclear localization of FOXC2, a transcription factor required for valvulogenesis. Further experiments revealed that the cell surface protein E-selectin was required for both of these responses. Our observations suggest that spatial gradients in WSS help to demarcate the locations of valve formation, and implicate E-selectin as a component of a mechanosensory process for detecting WSS gradients.

## Abstract (web summary)

Using microfluidic systems, Michalaki et al show that lymphatic endothelial cells exposed to wall shear stress orient in the direction perpendicular to flow and show increased nuclear FOXC2 levels in a manner dependent on E-selectin, a transmembrane adhesion protein. These data provide insights into how lymphatic vessels respond to local flow-mediated mechanical cues.

## Introduction

The lymphatic system helps maintain fluid balance in the body by draining excess interstitial fluid from tissues and depositing it into the bloodstream. Inadequate lymphatic drainage following injury, disease, or cancer treatment can lead to lymphedema, a debilitating condition characterized by chronic tissue swelling and impaired immune responses^1–5^. Lymphatic valves, similar to venous valves, are necessary for unidirectional lymph flow and thus for the physiological function of the lymphatic system^6^. During mouse embryonic development, lymphatic valve formation occurs through distinct steps that include upregulation of PROX1 and FOXC2, two transcription factors required for valve formation, alignment of lymphatic endothelial cells (LECs) perpendicular to the flow direction, increased deposition of laminin α5, and nuclear localization of nuclear factor of activated T-cells cytoplasmic 1 (NFATc1)^7–9^.

Both the formation and maintenance of lymphatic valves are governed by fluid flow^9,10^. LECs experience wall shear stresses (WSSs) ranging from 0 to 12 dyn cm^−2^ in rat mesenteric prenodal lymphatics^11^, and up to 40 dyn cm^−2^ in models of lymphedema^12^. Importantly, LECs are proposed to be responsive to variations in flow patterns in both time and space. For example, prior work suggests that oscillatory flow may regulate the activity of the transcription factors FOXC2 and GATA2, both of which are implicated in valve initiation and maintenance^9,10,13^. Although less explored, it is thought that spatial patterns in fluid flow may also influence valve development. During embryonic development, lymphatic valves preferentially form near vessel branches, junctions, and constrictions, all sites that feature local spatial gradients in WSS (WSS gradients; WSSGs)^5,9,14,15^. Prior to valve formation, mesenteric LECs located at vessel constrictions upregulate the transcription factors PROX1 and FOXC2, align perpendicular to the flow direction, and show increased nuclear localization of NFATc1^7–9^. These and other observations have led to the hypothesis that WSSGs, may provide an important cue that helps to control the timing and location of valve formation^5,15^.

How exactly LECs may sense WSSGs remains poorly understood. Most previous studies have focused on how vascular endothelial cells (ECs) sense and respond to WSS magnitude or temporal dynamics^16–18^. These studies and others identified PECAM-1, VE-cadherin, VEGFR-2 and VEGFR-3 as components of a signaling pathway that converts the physical stimulus provided by WSS into downstream signaling via VEGFR and PI3K signaling^16,19–21^. G-protein-coupled receptors (GPCRs), such as bradykinin B2^22^ and sphingosine 1-phosphate receptor 1 (S1PR1), have also been implicated in the response of ECs to fluid flow^23,24^. Despite these and other studies, how exactly ECs, and LECs specifically, may sense spatial variations in WSS remains little examined, due in part to a lack of experimental tools for exposing (L)ECs to controlled gradients in WSS.

We develop a microfluidic device that generates spatial gradients in WSS similar to those found at sites of lymphatic valve formation. We observe that WSSGs induce human lymphatic microvascular endothelial cells (HLMVECs) to orient perpendicular to flow, and trigger an increase in the nuclear localization of FOXC2, phenotypes that recapitulate the early steps of valvulogenesis in vivo. In addition, we find that E-selectin is required for both HLMVEC perpendicular alignment to flow and increased nuclear localization of FOXC2. These results indicate that WSSGs may provide a key cue in setting the timing and location of valve formation in vivo and implicate E-selectin as part of a flow sensing complex in HLMVECs. Devices such as we describe may prove generally useful for in vitro reconstitution of the physical stimuli present within the lymphatic system, and most particularly at sites of valve formation.

## Results

### A gradient in WSS orients HLMVECs perpendicular to flow

**Fig. 1:**
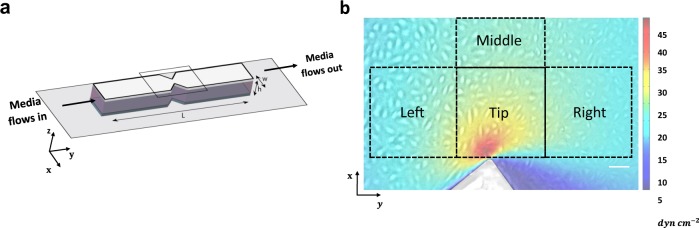
A constricted channel simulates spatial gradients in WSS found at sites of lymphatic valve formation. **a** We developed PDMS channels that have a sharp constriction from a uniform width (*w* = 5 mm) to a minimum width (*w*_min_ = 1.84 mm). The length of the channel (*L*) is 48.20 mm and the height 0.25 mm. **b** Superimposition of the WSS profile surrounding the constriction and a live-cell image showing HLMVECs adhering to the bottom surface of the channel. HLMVECs experience a sharp WSSG near the site of maximum constriction. Finite element simulation (COMSOL) shows the WSS profile for HLMVECs located near the constriction region. The flow is unidirectional in the *y*-direction. The WSS profile is symmetric across the *x* midline. Based on the flow direction in *y*, we binned data from the area around the constriction into four regions (400 × 400 μm): (Left) 18.5–30 dyn cm^−2^, (Tip) 9–50 dyn cm^−2^, (Right) 9–14.5 dyn cm^−2^, and (Middle) 22 dyn cm^−2^. Scale bar = 100 μm.

We sought to develop a microfluidic device that replicates the WSSGs near constrictions in the lymphatic vasculature, where lymphatic valves preferentially form^5,9,14,15^. We used soft-photolithography techniques to create channels with sharp constrictions in order to replicate the above flow environment (Fig. 1a and Supplementary Fig. 1 and 2; see Methods: Device Fabrication). Finite element modeling, which is highly accurate in the low Reynolds number regime (Fig. 1b; see Methods: Fluid Mechanics), revealed that WSS was locally concentrated at the area proximal to the constriction, creating a sharp WSSG with a maximum of 50 dyn cm^−2^ near the tips of the constriction, dropping to 22 dyn cm^−2^ in the middle of the constriction. This gradient in WSS approximately recapitulates that found near sites of lymphatic valve formation^5,9,11,25,26^. Although other geometries with various length scales and constriction types were tested (data not shown), we settled on the presented design because it facilitated reproducible fabrication and live-cell imaging while generating a sharp gradient in WSS similar to those found in the lymphatic system.

**Fig. 2:**
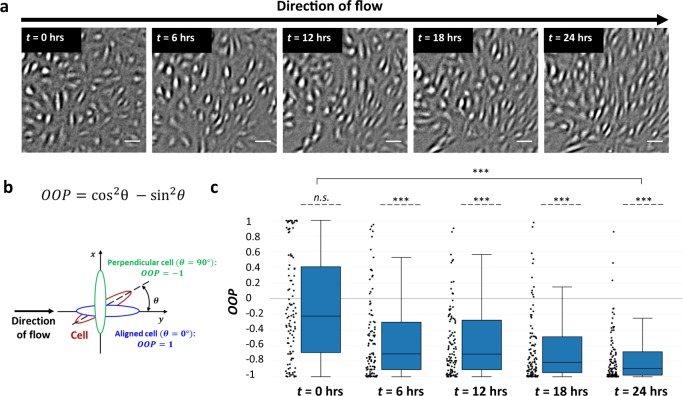
HLMVECs orient perpendicular to the flow direction in the presence of a gradient in WSS. **a** HLMVECs experiencing flow through a constriction turn perpendicular to the flow direction at the region of maximum WSS. Live-cell image shown is taken at the site of maximum constriction (Tip; WSS range = 9–50 dyn cm^−2^) (see Fig. 1b). The flow direction in each image is from left to right. Scale bar = 100 μm. **b** Schematic illustration of the orientation order parameter (OOP), where *θ* is the angle between the long axis of a cell and the direction of the flow. An OOP = 1 indicates alignment with the flow direction whereas OOP = −1 indicates perpendicular alignment with the direction of flow. **c** Quantification of orientation of HLMVECs in response to flow. Box-and-whisker plots showing the OOP of tracked HLMVECs at *t* = 0, 6, 12, 18, and 24 h. *N* = 100 cells, taken from two independent experiments. Dashed lines indicate a zero-median test while solid lines above the plots indicate a pairwise comparison for significance. Asterisks indicate that the compared distributions have statistically different medians, *p < 0.05, **p < 10^−3^ and ***p < 10^−7^. n.s. not statistically significant.

When first exposed to flow, HLMVECs presented no specific orientation on the surface of the channel near the constriction Tip (Fig. 2a). However, over the course of 24 h, HLMVECs at the Tip progressively aligned themselves perpendicular to the direction of flow at the region of maximum WSS (Fig. 2a and Supplementary Movie 1). Importantly, this alignment extended toward the Middle of the channel, well past the region with an appreciable WSSG (Supplementary Fig.3a). Thus, information contained in a local WSSG could propagate over longer distances, indicating that the alignment response was collective in nature.

To quantify HLMVEC alignment we defined an orientation order parameter (OOP) (Fig. 2b), where *θ* is the angle between the long axis of a cell and the direction of flow. The OOP ranges between −1 and +1, where an OOP value equal to −1 indicates alignment perpendicular to the flow direction while an OOP equal to +1 indicates alignment with the direction of flow. After 24 h of exposure to flow, HLMVECs in the region of the constriction oriented perpendicular to the flow (OOP< −0.7). In regions to the Left and Right of the Tip, where the WSSG is not as sharp, we found that there was no change of HLMVEC alignment (Supplementary Fig. 3b and c). In accordance with previous results^27–29^, cells exposed to spatially uniform WSS of 50 dyn cm^−2^ showed positive values of OOP > +0.7, indicating alignment with the direction of flow (Supplementary Fig. 4). We have previously observed that HLMVECs migrate against the direction of flow^24,30,31^. Here, we observed that, in the presence of flow, HLMVECs moved towards the constriction site (*x*-direction) but did not show migration in the direction of flow (*y*-direction) (Supplementary Fig. 5).

Together, these observations indicate that the presence of a sharp WSSG caused HLMVECs to align perpendicular to the direction of flow at the region of maximum WSS. These results suggest that the sharpness of the WSSG may be sufficient to trigger changes in cell alignment that accompany the formation of a lymphatic valve.

### Knockdown of E-selectin disrupts HLMVEC alignment perpendicular to flow

We screened for proteins that could potentially mediate the observed perpendicular alignment of cells specifically in response to a WSSG. In a previous study we noted that HLMVEC migration in response to flow depended on passage number. Motivated by this observation, we analyzed the transcriptome of HLMVECs as a function of passage number. Remarkably, only a small subset of gene transcripts was upregulated in cell passage numbers that showed the most robust perpendicular alignment to flow (Supplementary Fig. 6; Methods: Primary Cell Culture).

Of these potential hits, we focused on E-selectin (CD62E, SELE). E-selectin is an adhesion molecule that is highly expressed in ECs, and that has been shown to be responsible for the accumulation of leukocytes at sites of inflammation^32–35^. Also, it has been shown that in regions of disturbed flow in the vasculature, where there are WSSGs, ECs show a pronounced increase in the expression of E-selectin before other indications of atherosclerosis^16,36,37^. However, little is currently known about possible roles for E-selectin in sensing fluid flow.

**Fig. 3:**
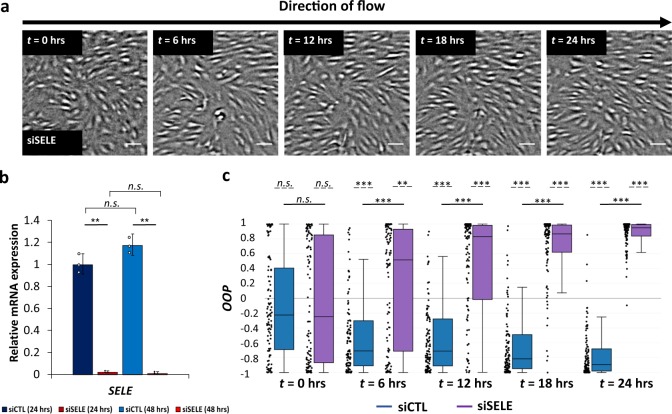
E-selectin is required for HLMVEC alignment perpendicular to flow. **a** HLMVECs treated with siRNA targeting E-selectin align with the flow direction after 24 h. Live-cell image are taken at the site of maximum constriction (Tip; WSS range = 9–50 dyn cm^−2^) (see Fig. 1b). The flow direction in each image is from left to right. Scale bar = 100 μm. **b** Relative *E-selectin* mRNA expression (referenced to scrambled siRNA control) for HLMVECs after 24 h and 48 h (blue: scrambled siRNA, siCTL; red: siRNA targeting E-selectin, siSELE), in the absence of flow. *N* *=* 7.5 × 10^4^ cells, taken from three independent experiments. Data points represent the results from independent experiments. Error bars represent the population standard deviation, propagated from the standard deviations of individual measurements **c** OOP of tracked HLMVECs at *t* = 0, 6, 12, 18, and 24 h treated with siCTL (blue) and siSELE (purple). *N* = 100 cells, taken from two independent experiments. Dashed lines indicate a zero-median test while solid lines above the plots indicate a pairwise comparison for significance. Asterisks indicate that the compared distributions have statistically different medians, *p < 0.05, **p < 10^−3^ and ***p < 10^−7^. n.s. not statistically significant.

We performed a transient siRNA knockdown of E-Selectin (siSELE; Fig. 3b), and subjected the resulting cells to flow in the constricted channel. Knockdown efficiency was ~98% as quantified using qPCR (Fig. 3b), though we were unable to quantify knockdown at the protein level owing to high nonspecific staining, in our hands, with commercially available E-selectin antibodies. After 24 h, the majority of HLMVECs treated with siCTL aligned perpendicular to the flow direction (OOP < −0.7) whereas HLMVECS treated with siSELE aligned parallel to the direction of flow (OOP > +0.8) (Fig. 3a and c). For the Left and Right regions of the channel, we found that both control cells and HLMVECs treated with siSELE were aligned parallel to the direction of flow (Supplementary Fig. 7a and b).

To determine whether knockdown of E-selectin disrupted HLMVEC alignment only in the presence of a WSSG, we quantified the alignment of HLMVECs treated with siSELE while they were exposed to uniform WSS equal to 50 dyn cm^−2^. We found that for both siCTL-treated cells and siSELE-treated cells, HLMVECs were initially randomly oriented and progressively aligned parallel to the direction of flow (OOP > + 0.7) (Supplementary Fig. 4). These results suggest that E-selectin is specifically required for HLMVECs to sense gradients in WSS, but not WSS.

### Increased nuclear FOXC2 in response to a WSSG is disrupted by E-selectin knockdown

**Fig. 4:**
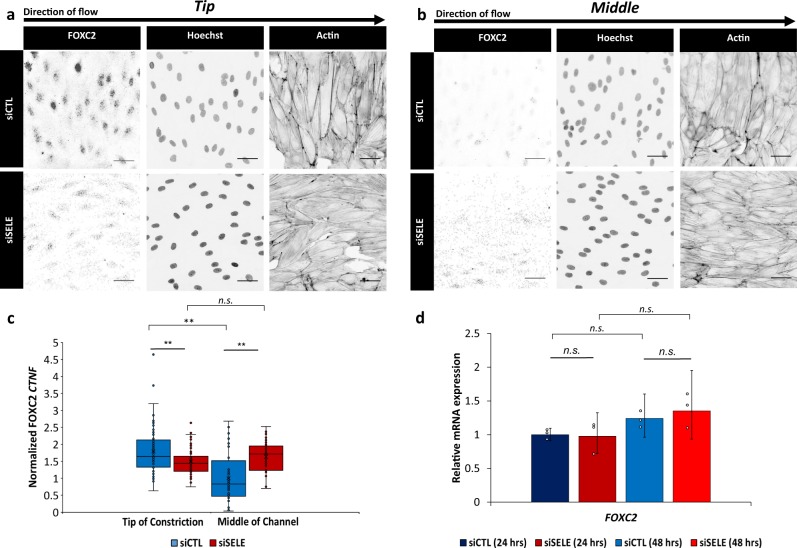
FOXC2 subcellular localization is WSSG dependent. **a, b** Immunofluorescence micrographs of FOXC2, Hoechst, and Actin for HLMVECs treated with scrambled siRNA (siCTL) and siRNA targeting E-selectin (siSELE) at the **(a)** Tip of the constriction (WSS range = 9–50 dyn cm^−2^) and **(b)** Middle of the channel (WSS = 22 dyn cm^−2^). HLMVECs were exposed to flow for 24 h with a maximum WSS of 50 dyn cm^−2^. The flow direction in each image is from left to right. Scale bar = 50 μm. **c** Normalized corrected nuclear fluorescence (CTNF) of FOXC2 at the Tip of the constriction and the Middle of the channel (WSS = 22 dyn cm^−2^) for HLMVECs treated with siCTL (blue) or siSELE (red). *N* = 80 cells for Tip and *N* = 40 cells for Middle, taken from two independent experiments. Error bars represent standard error on the mean. **d** Relative *FOXC2* mRNA expression (referenced to scrambled siRNA control) for HLMVECs after 24 h and 48 h (blue: scrambled siRNA, siCTL; red: siRNA targeting E-selectin, siSELE), in the absence of flow. *N* *=* 7.5 × 10^4^ cells, taken from three independent experiments. Data points represent the results from independent experiments. Error bars represent the population standard deviation, propagated from the standard deviaitons of individual measurements. Solid lines above the plots indicate a pairwise comparison for significance. Asterisks indicate that the compared distributions have statistically different medians, *p < 0.05, **p < 10^−3^ and ***p < 10^−7^. n.s. not statistically significant.

We subsequently investigated whether our findings correlated with in vivo studies showing that future lymphatic valve cells display an increase in nuclear FOXC2 and PROX1^5,7,9,10,38–42^. Immunofluorescence images at the Tip region revealed increased nuclear localization of FOXC2 compared with the Middle of the channel (Fig. 4, Supplementary Fig. 8). In contrast, nuclear levels of PROX1 were close to constant regardless of the examined region (Supplementary Fig. 9). These results demonstrate that FOXC2 nuclear localization is WSSG-controlled, while PROX1 nuclear localization is not responsive to WSSG.

Next, we sought to determine the effect of a transient siRNA knockdown of E-selectin on the nuclear localization of FOXC2. Knockdown of E-selectin decreased nuclear FOXC2 in cells near the constriction Tip relative to siCTL cells (Fig. 4b). Further, unlike in control cells, nuclear FOXC2 levels were similar for siSELE cells near the Tip of the constriction, to the Left and Right of the constriction, and in Middle of the channel, consistent with a loss in the ability of the siSELE cells to detect WSSGs (Fig. 4c, Supplementary Fig. 8c). Importantly, siSELE did not alter nuclear FOXC2 levels in the absence of flow (Supplementary Fig. 8c). These observations suggest that, in the model system studied here, FOXC2 nuclear localization is regulated by the presence of WSSGs in a manner that requires E-selectin.

To evaluate whether signaling involving E-selectin regulated FOXC2 at the transcriptional level, we determined the expression levels of *FOXC2* mRNA in the absence of flow for HLMVECs treated with scrambled siRNA and siRNA targeting E-selectin using qPCR. We found that there was no difference in the expression of *FOXC2* mRNA (Fig. 4d). Following the same analysis for *PROX1* mRNA, we showed that there was also no difference in its expression for HLMVECs treated with scrambled siRNA and siRNA targeting E-selectin in the absence of flow (Supplementary Fig. 9c).

In total, these findings demonstrate that the level of nuclear FOXC2 is sensitive to WSSGs, and that FOXC2 subcellular localization is regulated at least in part via a mechanism that requires E-selectin. In contrast, neither E-selectin nor WSSGs affected nuclear PROX1 levels.

## Discussion

In vivo studies demonstrate that murine mesenteric lymphatic valves preferentially form near constrictions, where ECs are subjected to WSSGs^5,9,43,44^. In addition, oscillatory flow plays an important role in valve maintenance, and the absence of flow leads to valve degeneration^9,10^. In this study, we found that the presence of a WSSG could yield long-range patterning of HLMVECs, namely alignment perpendicular to the direction of flow at the region of maximum WSS, in a manner that recapitulates cell alignment at sites of valve formation (Fig. 2). Interestingly, ECs also align perpendicular to flow in mature valves, suggesting that the cue provided by WSSGs may also contribute to valve maintenance^7,9^. Human adults seem to be unable to regenerate lymphatic valves in the case of damage^7,8,14^. Elucidating the underlying commonalities between valve formation and maintenance provides a potentially fruitful path forward in understanding how to encourage regeneration in the lymphatic system.

We observed that FOXC2 subcellular localization is WSSG dependent (Fig. 4), in agreement with the upregulation of FOXC2 observed at sites of valve formation in vivo^5,7,9,39–41^. On the other hand, under sufficiently high magnitudes of WSS, PROX1 exhibited high nuclear localization regardless of the WSSG or WSS levels. This finding contrasts with the specific increase in nuclear PROX1 observed at sites of valvulogenesis in vivo, and could indicate the existence of additional cues that help pattern valve formation^5,9^.

ECs in regions of the vasculature subject to disturbed flow, which feature WSSGs, show an increase in E-selectin expression before other indications of atherosclerosis^16,36,37^. While E-selectin has been shown to fulfill prominent functions in the blood vasculature, relatively less is known about its functions in the lymphatic vasculature. In our study, E-selectin was required for HLMVEC reorientation in response to a WSSG (Fig. 3c) but had no observable effect on alignment when cells were subjected to uniform WSS (Supplementary Fig. 4). In addition, HLMVECs treated with siRNA targeting E-selectin showed lower FOXC2 nuclear localization only in the presence of a gradient in WSS, implying that E-selectin may function in a WSSG-dependent manner. Future studies are required to determine how signal transduction involving E-selectin may influence FOXC2 subcellular localization. An important caveat is that E-selectin knockout mice are not reported to have phenotypes associated with lymphatic dysfunction, suggestive of a functional redundancy of E-selectin and other cell surface protein(s) during lymphatic development^45^. Identifying these proteins and their inferred, common downstream signaling pathway(s) activated in response to WSSGs provides an important avenue for future investigation.

Our observations suggest several additional avenues for future investigation. In particular, further studies will be required to determine if the molecular mechanisms that govern cell alignment in our system are the same as those that act in vivo. More broadly, in vivo studies have demonstrated that the presence of different flow regimes, namely laminar and oscillatory flow, lead to changes on LEC elongation and FOXC2 upregulation^9^. Examining how LECs, and ECs generally, respond to combined spatially and temporally varying flow, such as is found both in the lymphatic vasculature and at sites of atherosclerotic plaque formation, provides a promising avenue for future investigations^9,25,26^. In addition, we note that the present study examines only one constricted flow geometry. It is expected that other flow formats, such as constrictions of circular tube flow or simple asperities attached to tube walls will generate similar effects, but this remains to be tested. A dimensional analysis suggests that the WSSG present in vivo at sites of valve formation are likely to be of similar magnitude to the ones employed in our study, though the exact values will depend on vessel geometry and local flow rates. Additional experiments that vary the sharpness of the WSSG, and that recapitulate the size and three-dimensional geometry of the constrictions found in vivo may shed additional light on the question of how vessel geometry helps to dictate the timing and location of valve formation.

## Methods

### Device fabrication

Fabrication of the master: microfluidic devices were fabricated using photolithography and soft-photolithography techniques^46–48^. The device designs were created in AutoCAD (Autodesk, San Rafael, CA, USA) and the negative pattern of the device was printed onto a transparency photomask with a high-resolution printer (Artnet Pro, Inc., San Jose, CA, USA). Standard photolithography techniques were employed for wafer processing (Supplementary Fig. 1). A 4-inch silicon wafer (UniversityWafer, Inc., Boston, MA, USA) was cleaned by baking at 150 °C for 5 min in an oven. SU-8 photoresist was spin-coated on the clean silicon wafer at the thickness of 0.25 mm (SU-8 100, MicroChem Corp., Westborough, MA, USA) (Supplementary Fig. 1a). To achieve the desired thickness of the patterns the spinner was ramped up to 1000 r.p.m. at an acceleration of 100 r.p.m. s^−1^ for 45 s. After the SU-8 had been applied to the silicon wafer, it was soft-baked to evaporate the solvent and densify the film on a hot plate for 30 min at 65 °C and 90 min at 95 °C. Following the soft-bake, the mask was placed over the wafer and the assembly was exposed to ultraviolet light, causing the photoresist to photopolymerize where exposed (Supplementary Fig. 1b). After UV-exposure, a post-exposure bake was performed to selectively cross-link the exposed portions of the film. This bake was performed on a hot plate for 1 min at 65 °C and 20 min at 95 °C. Next, the wafer was developed in photoresist developer (SU-8 Developer, MicroChem Corp.) for 20 min, washed with isopropanol and gently dried using pressurized nitrogen gas (Supplementary Fig. 1c).

Fabication of polydimethylsiloxane devices (soft photolithography): microfluidic devices were made by replica molding using polydimethylsiloxane (PDMS; Sylgard 184, Dow Corning, Midland, MI, USA) using a 10:1 weight ratio of base to curing agent. Dust particles were first removed from the wafer using pressurized nitrogen gas. The wafer was then placed in an aluminum foil-lined dish to limit the required volume of PDMS and taped on a Petri dish (150 × 25 mm; 353025, Corning Life Sciences, Tewksburry, MA, USA). After degassing the mixed PDMS precursor solution in a vacuum chamber, it was poured on the wafer to the desired thickness (1–1.5 mm) and cured in an 80 °C oven for 90 min (Supplementary Fig. 1d). The polymerized PDMS device was then peeled off the silicon/SU-8 master (Supplementary Fig. 1e & f) and individual devices were separated using a scalpel. Inlet and outlet ports connecting to microfluidic channels were created using a standard 4 mm biopsy punch (Integra^TM^ Miltex^TM^ Biopsy Punches with Plunger System, Thermo-Fisher Scientific, Waltham, MA, USA). Before using the devices for cell culture, small particles were removed from the PDMS by applying and detaching Scotch tape. Next, the PDMS devices were placed in a glass beaker with deionized water and autoclaved at 121.1 °C using a wet cycle (20 min sterilization). The sterile devices were placed in a clean pipette tip box and autoclaved at 121.1 °C using a dry cycle (20 min sterilization and 15 min dry). Finally, the PDMS devices were dried in an oven at 80 °C overnight.

Cleaning glass coverslips: rectangular glass coverslips (24 × 60 mm, thickness: 0.15 mm; 22-266882, Thermo-Fisher Scientific) were air-dusted with pressurized nitrogen gas to remove dust particles from their surface. The coverslips were placed in a clean pipette tip box and autoclaved at 121.1 °C using a dry cycle (20 min sterilization and 15 min dry). They were dried in an oven at 80 °C overnight.

Device assembly: sterile PDMS devices (channel side facing up) and glass coverslips were plasma treated (PDC-32G, Harrick Plasma, Ithaca, NY, USA) for 5 min (Supplementary Fig. 2a). The devices and coverslips were immediately removed from the plasma treatment and bonded (Supplementary Fig. 2b). To assure proper bonding, the devices were softly pressed together with a sterile pair of tweezers, avoiding pressure on the channel area. The surface treatment renders the PDMS surface and coverslip reactive, resulting in an irreversible bond and preventing leaks^49^.

0.2% gelatin coating of the microchannels: we filled the microfluidic devices with 150 μL of surface coating solution. For HLMVECs culture, we pre-coated the channels with 0.2% gelatin (G1393, Sigma-Aldrich, St. Louis, MO, USA) dissolved in phosphate buffer saline (PBS; 10010023, Life Technologies) at 37 °C for 4 h (Supplementary Fig. 2c). Then, the gelatin solution was removed, the devices were filled with 150 μL of sterile DI water and washed 5 times using a micropipette. Excess surface coating solution can cause cell damage. Finally, all the channels were completely aspirated (Supplementary Fig. 2d).

Surface hydrophobicity restoration: in order to reassure proper cell growth, the devices should have their surface hydrophobicity restored^50^. To do so, the coated devices were placed in a sterile dish and directly in an oven at 80 °C for at least 24 h. Then, the microfluidic devices were filled with 150 μL of cell medium and stored in an incubator at 37 °C for at least 30 min before the seeding of the cells (Supplementary Fig. 2e). After this step, the microfluidic devices were ready to use.

### Fluid mechanics

Finite element analysis for WSS determination: numerical calculations were performed on the full Navier-Stokes equations using the commercial finite element analysis package COMSOL Multiphysics (COMSOL, Inc., Palo Alto, CA) to derive the WSSs in the constricted channel. Below, *z* = 0 refers to the channel’s bottom surface, where the imaged cells reside. We assumed incompressible, time-invariant and laminar flow. A two-dimensional axisymmetric geometry (Cartesian coordinates) was chosen and we took advantage of the symmetry condition across the *x* midline; analysis was performed for half of the constricted channel. The flow was unidirectional in the *y*-direction. To precisely simulate the curvature at the tip of the constriction as it was found on the mold of the constricted channel, we assumed a radius of curvature equal to 20 μm. The no-slip condition was applied to the walls of the constricted channel. At the inlet of the device, where fluid was inserted into the channel, the flow rate was specified as laminar flow in volumetric units (mL min^−1^). The pressure was set to ambient conditions at the outlet boundary. Lagrange quadratic shape functions were used to approximate the solution to the differential equations on each mesh element. The mesh was refined until the numerical values of the WSS changed by less than 1%. The hexahedral elements (rectangular prisms) elongated in the flow direction were used to decrease the model size in the fluid space. Reynolds numbers were calculated using the length of the channel as the length scale. All Reynolds numbers were in the laminar flow regime such that the FEA is sufficient to provide a faithful simulation of the experimental flow field.

### Primary cell culture

Endothelial cell culture: primary human lymphatic microvascular endothelial cells (HLMVECs; CC-2810) were purchased from Lonza Corporation (Walkersville, MD, USA) and cultured in EGM-2 basal medium (CC-3156, Lonza Corporation) containing supplements and growth factors (CC-4147, Lonza Corporation) that include 5% fetal bovine serum, hEGF, VEGF, hFGF-B, R3-IGF-1, hydrocortisone, and ascorbic acid. 50 units mL^−1^ of penicillin and 50 mg mL^−1^ streptomycin (15140–122, Life Technologies, Carlsbad, CA, USA) were added to the medium. Cells used for experiments were between passages 6 and 8. Three to five days before the flow experiment, depending on the desired initial confluency, cells were plated onto the channels. HLMVECs were plated at 7.5 × 10^4^ cells per channel and incubated at 37 °C and 5% CO_2_.

Prior to imaging, the EGM-2 medium was exchanged to Leibovitz’s L-15 medium (21803-027, Life Technologies) to allow for imaging independent of CO_2_. The L-15 medium included 5% FBS, the endothelial growth factor kit from Lonza (CC-4147, Lonza Corporation), 50 units mL^−1^ of penicillin and 50 mg mL^−1^ streptomycin (Life Technologies). HLMVEC experiments were performed with cells at surface coverage (fraction of the glass coverslip covered by cells) of greater than 95% and confluency (fraction of maximum cell density) of 80% to ensure sufficient contact with neighboring cells.

SiRNA experiments: small interfering RNA (siRNA) knockdown experiments were performed using the Lipofectamine® RNAiMAX Transfection Reagent (13778030, Thermo-Fisher Scientific) to deliver siGENOME SMARTpool SELE siRNA (5 nmol; 6401, GE Dharmacon, Lafayette, CO, USA). Scrambled siRNA (Negative Control of siRNA Duplex; 027210, QIAgen, Hilden, Germany) was used for both quantitative real-time PCR and flow experiments as a negative control to verify that siRNA delivery did not adversely affect the HLMVECs. Delivery of siRNA was performed based on a standardized protocol detailed by Life Technologies: 1 day prior to the flow experiment, siRNA and Lipofectamine dilutions in serum-free Opti-MEM^®^ (11058021, Thermo-Fisher Scientific) were prepared separately, mixed and incubated at room temperature for 20 min. The resulting solution was added dropwise to HLMVECs (approximately 80% confluency) plated on the constricted channel in EGM-2 medium and incubated at 37 °C for 24 h. After incubation, the EGM-2 medium with Lipofectamine/siRNA was exchanged with L-15 and the flow experiment was performed. siRNA knockdown experiments were also performed in the parallel plate channels.

Quantitative real-time PCR: RNA was isolated and purified using the GENEjet RNA Purification Kit (#K0731, Thermo-Fisher Scientific). RNA was converted to cDNA using the Applied Biosystems High Capacity Reverse Transcription Kit (4368814, Thermo-Fisher Scientific). Quantitative real-time PCR was performed through use of the Applied Biosystems Step One Plus Real Time PCR System (Thermo-Fisher Scientific) with Power SYBR® Green PCR Master Mix (4367659, Thermo-Fisher Scientific). To determine the relative mRNA expression levels, two normalization factors were used: the gene *ActB* and the negative control samples that had been treated with scrambled siRNA. The *ActB* gene served as a housekeeping gene and the scrambled siRNA samples as a reference baseline by which relative *SELE*, *FOXC2*, and *PROX1* mRNA expression levels were compared. *ActB* primers used were: Forward 5’-TTCTACAATGAGCTG CGTGTG-3’ and Reverse, 5’-ATCACAATGCCAGTGGTACG-3’. *Sele* qPCR primers used were: Forward, 5’-GGCAGTGGACACAGCAAATC-3’ and Reverse, 5’-TGGACA GCATCGCATCTCA-3’. *Foxc2* primers used were: Forward 5’-CTACAGCTACATCGCGCTCATCA-3’ and Reverse, 5’-ACTGGTAGATGCCGTTCAAGGTG-3’. *Prox1* primers used were: Forward 5’-CCCAGGACAGTTTATTGACCGA-3’ and Reverse, 5’-GGTTGTAAGGAGTTTGGCCCAT-3’. All trials were run in triplicate.

Immunofluorescence cell staining: after the flow experiments, the samples were fixed with 4% paraformaldehyde (Fluka, Sigma-Aldrich, St. Louis, MO, USA) in PBS for 15 min at room temperature. After two washes in PBS (2 min each), the cells were permeabilized with 0.5% Triton X-100 (X100, Sigma-Aldrich) in PBS (v%) for 10 min at room temperature. After two washes in PBS (5 min each), the cells were blocked with 1% bovine serum albumin (BSA; A9418-5G, Sigma-Aldrich) in PBS (w%) and put on a rocking table for 90 min. HLMVECs were stained with mouse anti-FOXC2 antibody (0.5 mg mL^−1^ stock solution in PBS; ab55004, Abcam, Cambridge, UK) and rabbit anti-PROX1 antibody (1 mg mL^−1^ stock solution in PBS; ab101851, Abcam) at 1:500 dilution in 1% BSA solution overnight at 4 °C. The next day, the devices were rinsed three times in PBS (5 min each) and incubated in the dark (samples covered with aluminum foil) with Alexa Fluor® 488-conjugated anti-mouse IgG (2 mg mL^−1^, Cell Signaling Technology, Danvers, MA, USA) and Alexa Fluor® 647-conjugated anti-rabbit IgG (2 mg mL^−1^, Cell Signaling Technology) at 1:1000 dilution for 90 min at room temperature. After one rinse in PBS (5 min), actin filaments and cell nuclei were labeled for F-actin (2.5 mg; ActinRed^TM^ 555, Life Technologies) and with Hoechst 34580 (5 mg stock solution in water; H21486, Life Technologies), respectively, at 1:100 dilution for 15 min at room temperature, followed by two rinses in PBS (5 min each). The fluorescently stained samples were visualized on a Nikon Ti (Nikon Corporation, Tokyo, Japan) microscope for fluorescence imaging using a 20× air objective, and imaged using an Andor Neo camera.

Transcriptome analysis: RNA was isolated and purified using the GENEjet RNA Purification Kit (#K0731, Thermo-Fisher Scientific). RNA was converted to cDNA using the Applied Biosystems High Capacity Reverse Transcription Kit (4368814, Thermo-Fisher Scientific). HLMVEC cDNA samples were provided for various passage numbers, i.e. passage 4 (P4), passage 7 (P7), and passage 10 (P10), in triplicate. cDNA was then provided to the Stanford Functional Genomics Facility where the samples were subsequently run on an Affymetrix GeneChip® System 3000 (Thermo-Fisher Scientific).

### Flow experiments

Experimental setup: the channel was mounted on a *xy* translation stage (H101A ProScan^TM^ Stage, Prior Scientific, Inc., Rockland, MA, USA) over an inverted microscope. The existence of an inlet and outlet at the ends of the device allowed recirculation of the flow medium during the flow experiment. The cell culture medium was recirculated using a 9-roller dampened peristaltic pump (Idex, Oak Harbor, WA, USA). The inlet and outlet ports were placed far away from the center region of the channel preventing any perturbation of the local flow environment.

The experiments were performed with a Nikon TE microscope for brightfield imaging using a 4× air objective with a 1.5× tube lens Nikon objective and a Flea 3 camera (Point Grey, BC, Canada). The microscope was equipped with a temperature control chamber to maintain the temperature at 37 °C.

Preparation for flow experiments: prior to the flow experiment, the tubing was washed twice (5 and 15 min washes) in sterile water and PBS, consecutively. Following these four washes, the tubing washed in L-15 for 30 min. Meanwhile, the EGM-2 medium was removed from the channel and replaced with L-15. Then, the channel positioned on top of the *xy* translation stage. The flow rate for all the experiments in the constricted channels was 3.8 mL min^−1^, corresponding to a maximum WSS of 50 dyn cm^−2^ near the constriction. Parallel plate experiments were performed with a uniform WSS of 50 dyn cm^−2^ using a flow rate of 4.27 mL min^−1^. All flow experiments were performed for 24 h. Following the experiment, the tubing was cleaned by applying two washes in water of 5 min and 15 min each, followed by one wash in 70% ethanol for 30 min. Control experiments were performed using an identical culture of HLMVECs imaged under the same conditions, but in the absence of flow.

### Data analysis

Determination of cell alignment: brightfield movies were processed with Fiji software^51^. All image files were bandpass filtered in Fiji using high- and low-frequency cutoffs at 2 and 20 pixels (approximately 2.5 and 25 μm). The cells were binned based on their initial position regarding the center of the constriction (or device) (400 × 400 μm regions). Cell alignment was tracked in time intervals of 6 h for the entire duration of the flow experiment, e.g. 0, 6, 12, 18, and 24 h, using the OrientationJ Measure plugin in Fiji for 50 cells per binned region. Using that plugin, the angle *θ* was measured between a specific cell and the direction of flow. An OOP was determined using the calculated angle *θ*. The OOP ranged between −1 and +1, where a value of OOP equal to −1 indicated an alignment perpendicular to the flow direction whereas a value equal to +1 an alignment with the direction of flow. The alignment of HLMVECs was determined for duplicate data sets (100 cells in total per trial) and graphed as box-and-whisker plots using Plotly (plot.ly).

Determination of cell migration: brightfield movies were processed in the same way as our previous publications^24,30^. Movies were processed with Fiji software and analyzed using custom Matlab routines. All image files were bandpass filtered in Fiji using high- and low-frequency cutoffs at 2 and 20 pixels (approximately 2.5 and 25 mm). The cells were binned based on their initial position regarding the center of the constriction (400 × 400 μm regions). Cell migration was tracked for 24 h using the Manual Tracking plugin in Fiji for 50 cells per binned region. Cellular *x*- and *y*-positions were determined every 20 min and input into a custom Matlab script. The *x*- and *y*- displacements were calculated ranging between negative and positive values, where negative values indicate downstream migration while positive values indicate upstream migration. The migration of HLMVECs was determined for duplicate data sets (100 cells in total per trial) and graphed on box-and-whisker plots using Plotly (plot.ly).

Calculation of nuclear fluorescence intensity: immunofluorescence micrographs cells stained for FOXC2, PROX1, Actin and DNA (Hoecsht dye) were processed with Fiji software. The Hoecsht image was used as the reference to develop a nuclear mask. This was performed by tracing an oval around the cell nucleus, transferring this mask to either the FOXC2 or PROX1 channels, and tabulating the respective Area and Integrated Density parameters from ImageJ. Based on that acquired measurement, the normalized corrected nuclear fluorescence (Normalized CTNF) for either FOXC2 or PROX1 was determined using the following equation^52^:

$${\rm{Normalized}}\,{\rm{CTNF}} = 	\frac{1}{{{\rm{Area}}}}\,[{\rm{Integrated}}\,{\rm{Density}} \\ 	- ({\rm{Area}}\, \times \,{\rm{Mean}}\,{\rm{Gray}}\,{\rm{Value}}{\kern 1pt} {\rm{of}}\,{\rm{Background}})].$$

The background fluorescence intensity was determined through tracing an area analogous to a cell nucleus and determining the Mean Gray Value of Background. The corrected nuclear FOXC2 and PROX1 intensities were calculated for duplicate data sets (40 cells in total per trial).

Statistics and reproducibility: tests for pairwise equal median statistical significance (i.e. tests the null hypothesis that the medians of the populations from which two samples are drawn are identical) were performed using the Wilcoxon rank sum test, while tests for zero-median statistical significance (i.e. tests the null hypothesis that the medians of the populations from which two samples are drawn are equal to zero) of a data set were determined using the Wilcoxon ranked sign test. Unless otherwise specified, for all box-and-whisker plots, solid lines indicate the pairwise equal median comparison. In these plots, the solid line represents the median of the data set. Not significant (n.s.) indicates that the medians of the pair of distributions are statistically indistinguishable. For the OOP plots, dashed lines indicate results from the zero-median test, where n.s. indicates that the distribution has a median statistically indistinguishable from zero. Statistical significance is denoted by asterisks above the significance bar. A single asterisk indicates *p* < 0.05, whereas two or three asterisks indicate *p* < 10^–3^ or *p* < 10^–7^, respectively. All analyses shown are for duplicate data sets, where two independent flow experiments were performed from HLMVECs cultured in different batches (biological samples). Different biological samples originated from different stocks of primary cells. For OOP plots *N* = 100 cells were analyzed while for the Normalized CTNF plots (FOXC2 and PROX1) *N* = 80 (or 40) cells, taken from two independent experiments.

### Reporting summary

Further information on research design is available in the Nature Research Reporting Summary linked to this article.

## Supplementary information

Supplementary Information

Description of Additional Supplementary Files

Supplementary Data 1

Supplementary Movie 1

Reporting summary

## Data Availability

All data generated or analyzed during this study are included in this published article (and its Supplementary Information files). The source data underlying Figs. 2–4 and Supplementary Figs. 3–9 are shown in Supplementary Data 1.

## Code availability

The codes that used for data analysis of this study are available from the corresponding author (A.R.D.) upon reasonable request.
