# Supplementary material for: Perpendicular alignment of lymphatic endothelial cells in response to spatial gradients in wall shear stress
Source: Commun Biol. 2020 Feb 6;3:57. doi: 10.1038/s42003-019-0732-8 (PMC7005002; doi:10.1038/s42003-019-0732-8)
Supplement: Supplementary file 1 — Supplementary Information [file 42003_2019_732_MOESM1_ESM.pdf]

Supplementary Figures

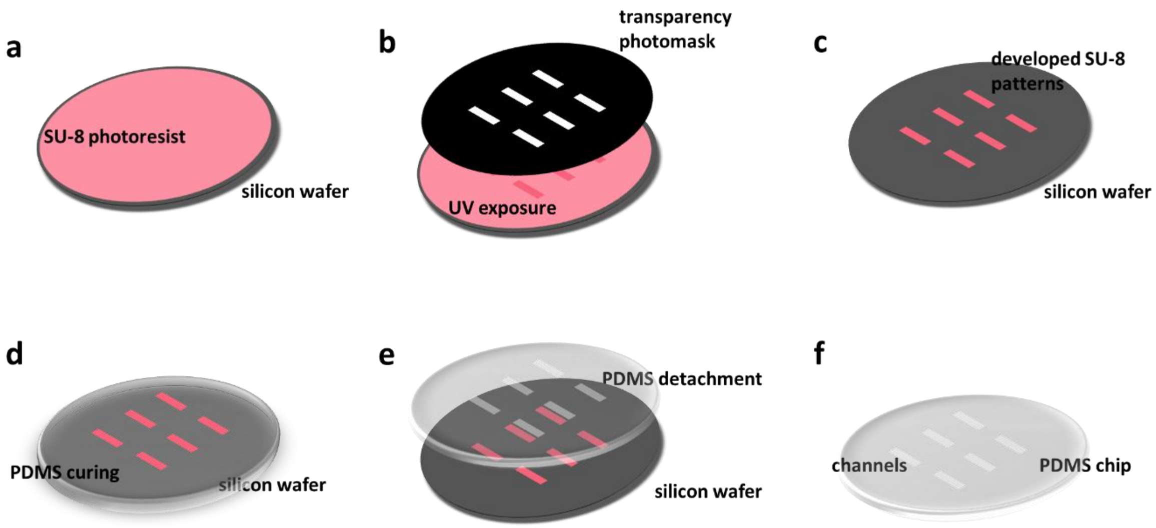

Supplementary Fig. 1 Microfluidic devices were fabricated using photolithography and soft-photolithography techniques. **(a-c)** Photolithography steps. **(d-f)** Soft photolithography steps. **(a)** SU-8 is spin coated and baked on a silicon wafer. **(b)** UV light cures exposed regions of the SU-8 layer. **(c)** The SU-8 is then baked, and the uncrosslinked regions are removed using developer solution. **(d)** PDMS precursor is poured on the wafer and cured. **(e)** Cured PDMS is peeled from the wafer. **(f)** The PDMS chip is ready to use for channel preparation (Supplementary Fig. 2).

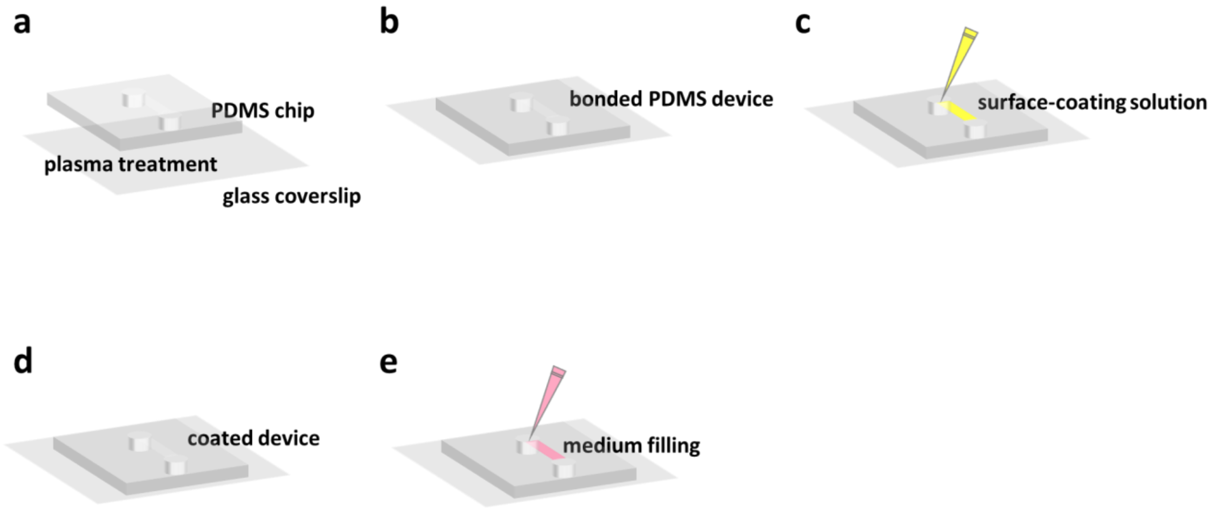

Supplementary Fig. 2 Preparation of channels for cell culture. **(a, b)** PDMS devices are attached to glass coverslips using atmospheric oxygen plasma treatment. **(c)** A surface coating solution (e.g. gelatin) is added to the channel, which is then placed in the incubator. **(d)** The coated channel is placed in an 80 °C oven for surface hydrophobicity restoration. **(e)** Channel(s) are filled with cell culture medium preparatory to cell seeding.

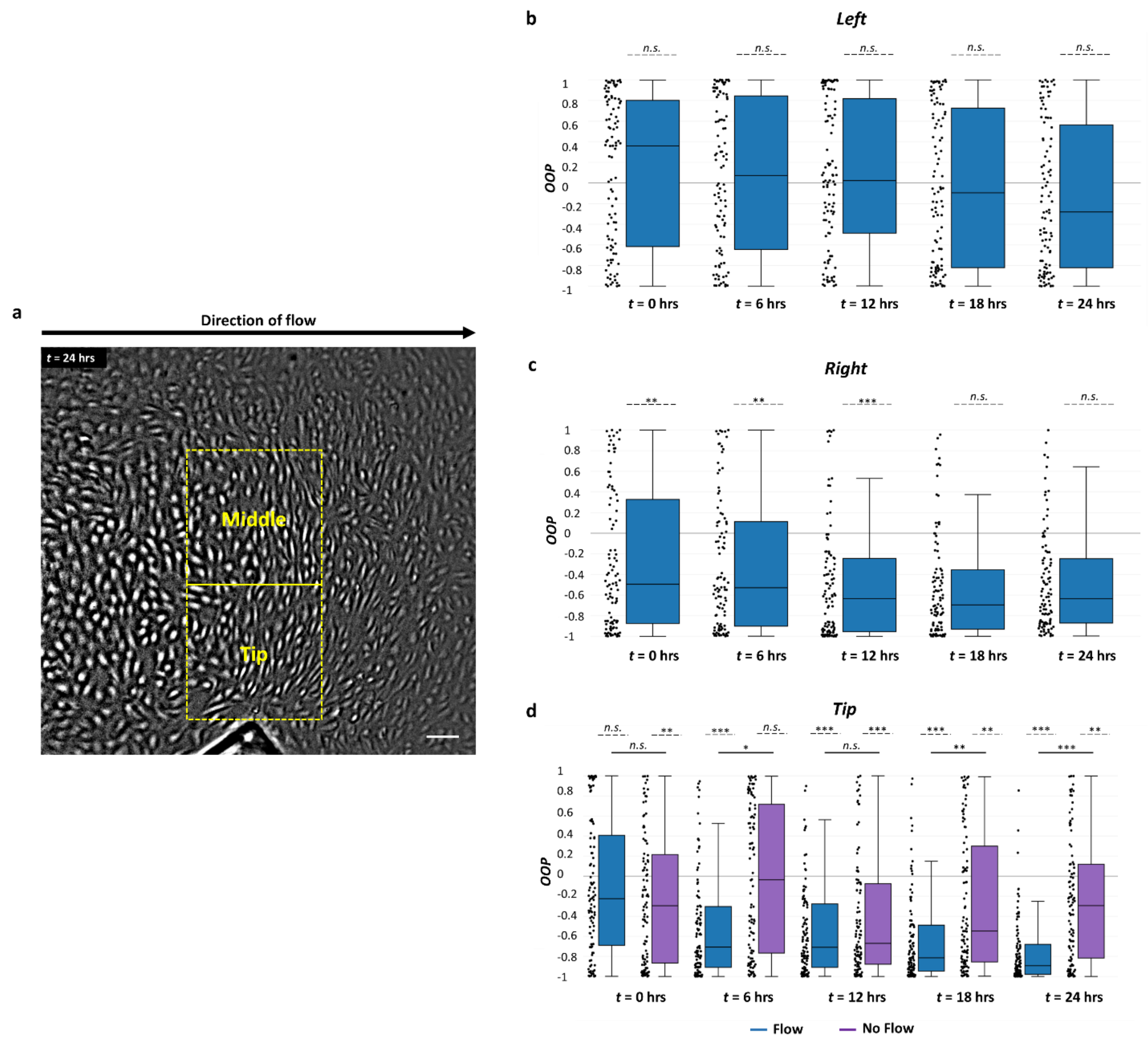

Supplementary Fig. 3 HLMVECs collectively orient perpendicular to the flow direction in the presence of a gradient in WSS. **(a)** HLMVECs experiencing flow through a constriction collectively turn perpendicular to the flow direction at the region of maximum WSS. (*Tip*: WSS range = 9 – 50 dyn cm^{-2} and *Middle*: WSS = 22 dyn cm^{-2}). The flow direction is from left to right. Scale bar = 100 μm. *OOP* of tracked HLMVECs at *t* = 0, 6, 12, 18, and 24 hrs. **(b)** *Left* (WSS range = 18.5 – 30 dyn cm^{-2}) and **(c)** *Right* (WSS range = 9 – 14.5 dyn cm^{-2}) regions. **(d)** Comparison between the presence and absence of flow for the *Tip* (WSS range = 9 – 50 dyn cm^{-2}) region (blue: flow; purple: no flow). *N* = 100 cells, taken from two independent experiments. Dashed lines indicate a zero median test while solid lines above the plots indicate a pairwise comparison for significance. Asterisks indicate

that the compared distributions have statistically different medians, $p^* < 0.05$, $p^{**} < 10^{-3}$ and $p^{***} < 10^{-7}$. *n.s.* (not statistically significant).

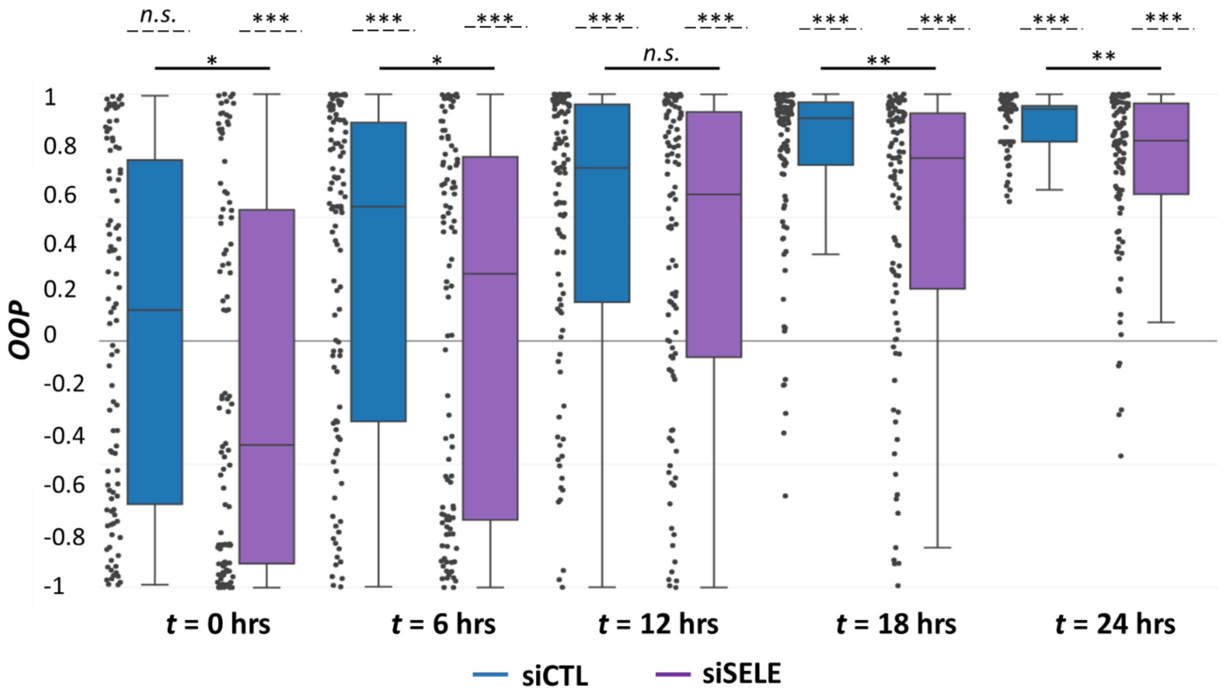

Supplementary Fig. 4 *OOP* of tracked HLMVECs at *t* = 0, 6, 12, 18, and 24 hrs subjected to uniform WSS (WSS = 50 dyn cm^{-2}) (blue: scrambled siRNA, siCTL; purple: siRNA targeting E-selectin, siSELE). *N* = 100 cells, taken from two independent experiments. Dashed lines indicate a zero median test while solid lines above the plots indicate a pairwise comparison for significance. Asterisks indicate that the compared distributions have statistically different medians, $p^* < 0.05$, $p^{**} < 10^{-3}$ and $p^{***} < 10^{-7}$. *n.s.* (not statistically significant).

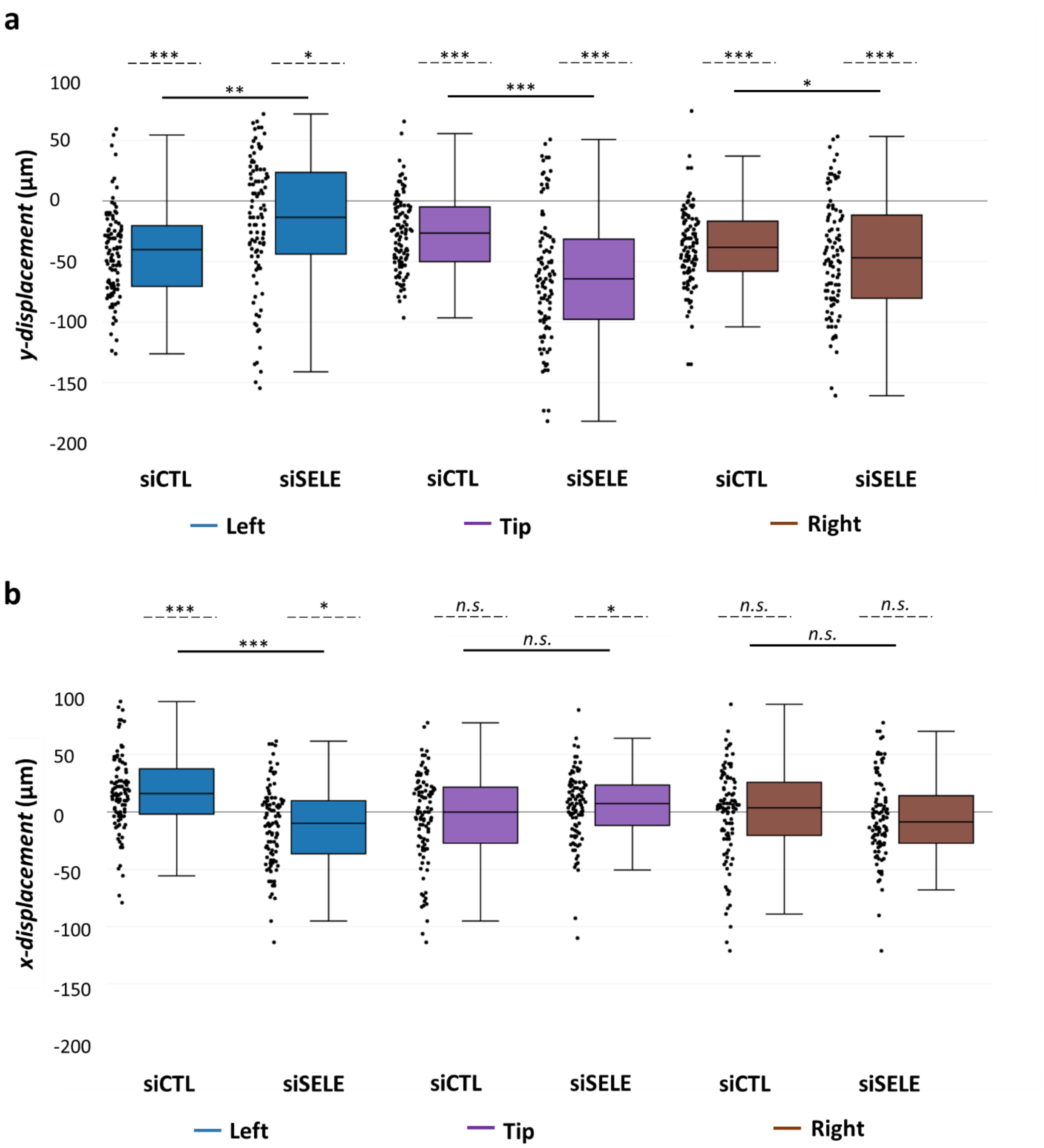

Supplementary Fig. 5 *y- and x- displacements*. **(a)** *y-* and **(b)** *x- displacements* for tracked HLMVECs in *Left* (WSS range = 18.5 – 30 dyn cm^{-2}; blue), *Tip* (WSS range = 9 – 50 dyn cm^{-2}; purple), and *Right* (WSS range = 9 – 14.5 dyn cm^{-2}; brown) regions. Positive *y-displacement* values indicate migration with the direction of flow while negative values migration against the direction of flow. Positive *x-displacement* values indicate upstream migration while negative values downstream migration. *N* = 100 cells, taken from two

independent experiments. Dashed lines indicate a zero median test while solid lines above the plots indicate a pairwise comparison for significance. Asterisks indicate that the compared distributions have statistically different medians, $p^* < 0.05$, $p^{**} < 10^{-3}$ and $p^{***} < 10^{-7}$. *n.s.* (not statistically significant).

a

Gene Name	Gene Symbol	P7-P4	P7-P10	Description
**E-Selectin**	SELE	4.89	3.95	Found in ECs, mediates cell adhesion to vascular wall
**Endogenous Retrovirus Group FRD Member 1**	ERVFRD-1	3.83	2.51	Human endogenous retroviral envelope protein 1
**F-Box Protein 32**	FBXO32	2.62	1.99	Muscle atrophy resistance transcription factor
**Phospholipase C Epsilon 1 (Anti Sense Strand)**	PLCE-AS1	2.29	3.71	Antisense strand of PLCε, converts signaling lipids for downstream Ca^{2+} signaling

b

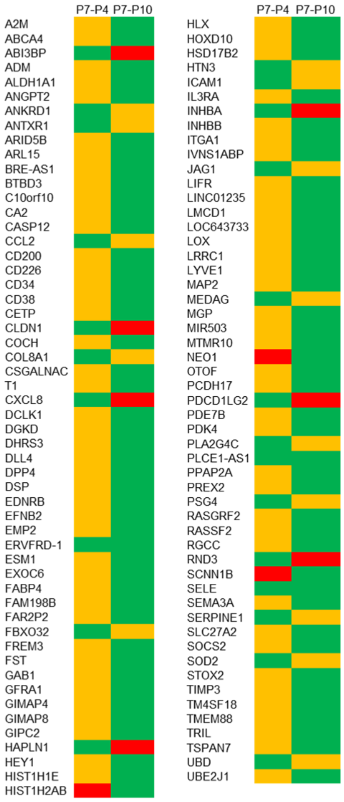

Supplementary Fig. 6 Whole transcriptome analysis for HLMVECs as a function of passage number. **(a)** Comparison among different cell passage numbers (P7 minus P4 and P7 minus P10) demonstrated that only a small subset of gene transcripts was upregulated in cell passage numbers (approximately P7) that showed the most robust perpendicular alignment to flow relative to both earlier (P4) and later (P10) passages. Note that mRNA samples were isolated from cells cultured in the absence of flow. **(b)** A more exhaustive presentation of the gene transcripts with the biggest differences among the various cell passage numbers. The absolute levels are compared among the different gene transcripts with the red color indicating the lowest (P7 minus P4 and P7 minus P10 < -2), yellow the intermediate (-2 > P7 minus P4 and P7 minus P10 > 2), and green the highest value of the set (P7 minus P4 and P7 minus P10 > 22).

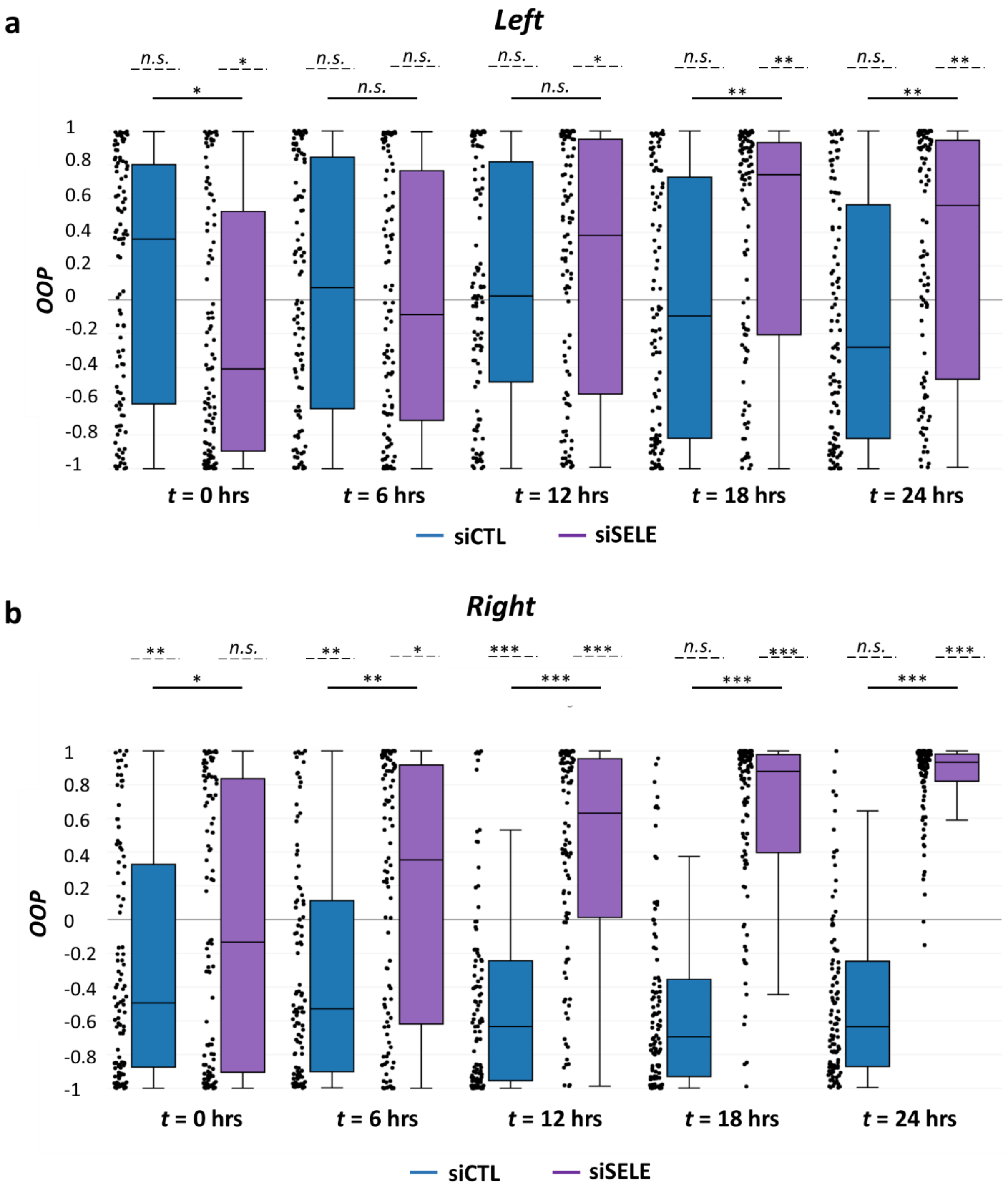

Supplementary Fig. 7 *OOP* of tracked HLMVECs at t = 0, 6, 12, 18, and 24 hrs. **(a)** *Left* (WSS range = 18.5 – 30 dyn cm^{-2}) and **(b)** *Right* (WSS range = 9 – 14.5 dyn cm^{-2}) regions (blue: scrambled siRNA, siCTL; purple: siRNA targeting E-selectin, siSELE). *N* = 100 cells, taken from two independent experiments. Dashed lines indicate a zero median test

while solid lines above the plots indicate a pairwise comparison for significance. Asterisks indicate that the compared distributions have statistically different medians, $p^* < 0.05$, $p^{**} < 10^{-3}$ and $p^{***} < 10^{-7}$. *n.s.* (not statistically significant).

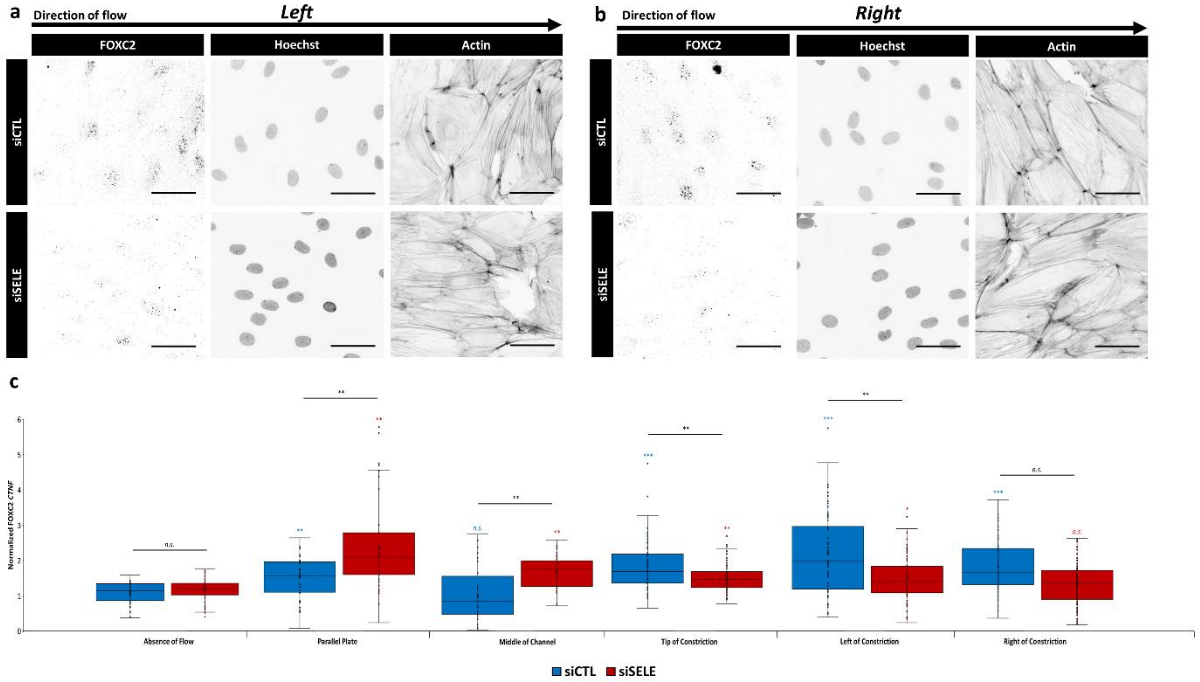

Supplementary Fig. 8 Immunofluorescence images of FOXC2, Hoechst, and Actin. HLMVECs treated with scrambled siRNA and siRNA targeting E-selectin for the **(a)** *Left* (WSS range = 18.5 – 30 dyn cm^{-2}) and **(b)** *Right* (WSS range = 9 – 14.5 dyn cm^{-2}) regions. The flow direction in each image is from left to right. Scale bar = 50 µm. **(c)** Normalized *CTNF* for FOXC2 for HLMVECs treated with scrambled siRNA (siCTL; blue) or siRNA targeting E-selectin (siSELE; red) for various flow conditions: *Parallel Plate* (WSS = 50 dyn cm^{-2}), *Middle* of the channel (WSS = 22 dyn cm^{-2}), *Tip* (WSS range = 9 – 50 dyn cm^{-2}), *Left* (WSS range = 18.5 – 30 dyn cm^{-2}), and *Right* (WSS range = 9 – 14.5 dyn cm^{-2}). *N* = 40 cells for absence of flow, *Parallel Plate*, and *Middle*, and *N* = 80 cells for *Tip*, *Left* and *Right*, taken from two independent experiments. One off-scale data point is not depicted. This is panel c: (*Parallel Plate* - siSELE) 9.04 a.u. Solid lines above the box-and-whisker plots indicate a pairwise comparison for significance while asterisks on top of the plots indicate comparison with the corresponding absence of flow condition (i.e. blue: with siCTL absence of flow; red: with siSELE absence of flow). Asterisks indicate that the compared distributions have statistically different medians, $p^* < 0.05$, $p^{**} < 10^{-3}$ and $p^{***} < 10^{-7}$. *n.s.* (not statistically significant).

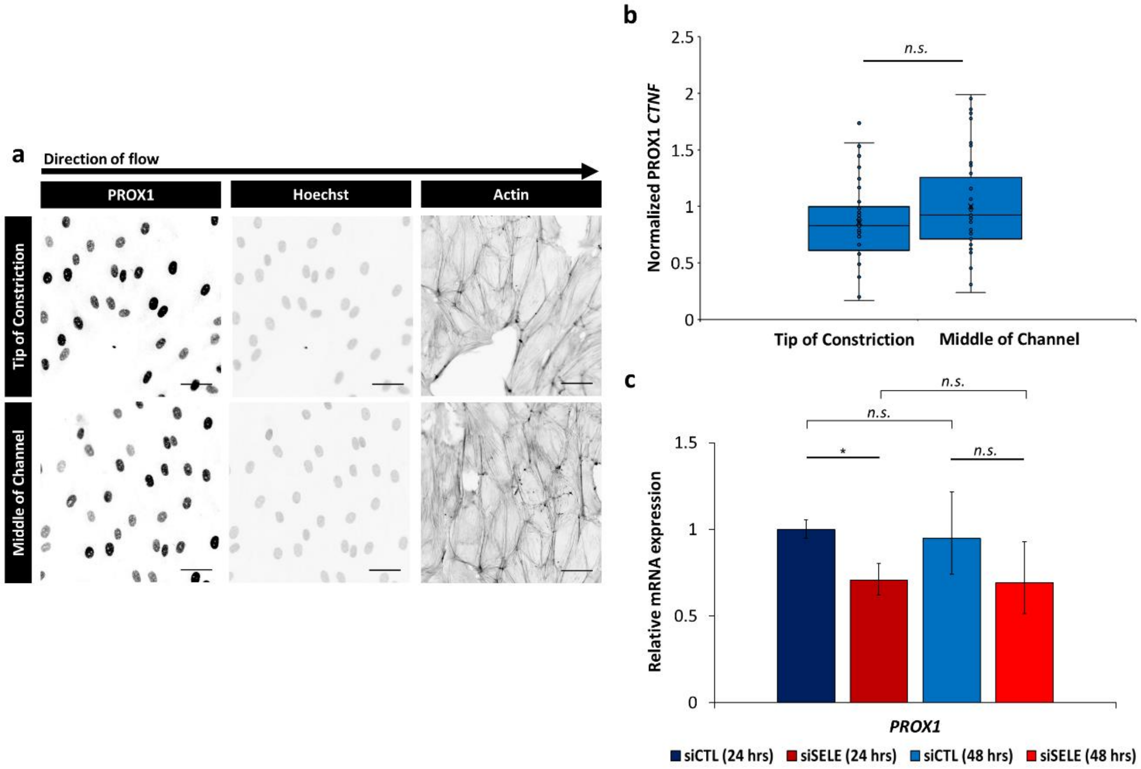

Supplementary Fig. 9 Nuclear localization of PROX1 in response to flow. **(a)** Immunofluorescence micrographs of PROX1, Hoechst, and Actin. HLMVECs exposed to flow for 24 hrs with a maximum WSS of 50 dyn cm^{-2}. The flow direction in each image is from left to right. Scale bar = 50 μm. **(b)** Normalized *CTNF* of PROX1 at the *Tip* of the constriction (WSS range = 9 – 50 dyn cm^{-2}) and the *Middle* of the channel (WSS = 22 dyn cm^{-2}) for HLMVECs. *N* = 40 cells, taken from two independent experiments. Error bars represent standard error on the mean. **(c)** Relative PROX1 mRNA expression (referenced to scrambled siRNA control) for HLMVECs after 24 hrs and 48 hrs in the absence of flow. Data for siCTL cells are in blue while data for siSELE are in red. Error bars represent population standard deviation. Solid lines above the plots indicate a pairwise comparison for significance. Asterisks indicate that the compared distributions have statistically different medians, $p^* < 0.05$, $p^{**} < 10^{-3}$ and $p^{***} < 10^{-7}$. *n.s.* (not statistically significant).
