# Supplementary material for: Perpendicular alignment of lymphatic endothelial cells in response to spatial gradients in wall shear stress
Source: Commun Biol. 2020 Feb 6;3:57. doi: 10.1038/s42003-019-0732-8 (PMC7005002; doi:10.1038/s42003-019-0732-8)
Supplement: Supplementary file 2 — Description of Additional Supplementary Files [file 42003_2019_732_MOESM2_ESM.pdf]

Description of Additional Supplementary Files

File Name: **Supplementary Movie 1**
Description: HLMVECs experiencing flow through a constriction, recorded for 24 hrs. The flow direction is from left to right. Scale bar = 100 μm.

File Name: **Supplementary Data 1**
Description: All data generated or analyzed during this study are included in this published article (and its Supplementary Information files). The source data underlying Figs. 2–4 and Supplementary Figs. 3–9 are shown in Supplementary Data 1. In each tab of the spreadsheet, the raw data of each corresponding figure can be found.
